# Parental phubbing and mobile phone addiction among Chinese adolescents: a moderated mediation model

**DOI:** 10.3389/fpsyg.2024.1379388

**Published:** 2024-05-27

**Authors:** Shutao Ma, Xiaoyan Bi, Hongbo Cui, Yankun Ma

**Affiliations:** ^1^Department of Psychology, School of Education, Guangzhou University, Guangzhou, China; ^2^Guangzhou Xinsui School, Guangzhou, China

**Keywords:** Chinese adolescent, parental phubbing, mobile phone addiction, deviant peer affiliation, sensation seeking

## Abstract

It has been reported that parental phubbing is a significant predictor of mobile phone addiction (MPA) among adolescents. However, the mechanisms underlying this association remain largely unclarified. On the basis of the social learning theories and ecological systems, this study assessed the mediating effect of deviant peer affiliation and the moderating effect of sensation seeking in the association between parental phubbing and MPA among Chinese adolescents. A total of 786 Chinese adolescents (mean age = 13.17 years, *SD* = 1.35) completed the questionnaires anonymously about parental phubbing, MPA, deviant peer affiliation and sensation seeking. After controlling for study variables, deviant peer affiliation could partially mediate the association between parental phubbing and MPA among adolescents and this indirect path could be moderated by sensation seeking. Notably, the effect of deviant peer affiliation on MPA was more pronounced in adolescents with higher sensation seeking than in those with lower sensation seeking.

## 1 Introduction

As of June 2023, the number of mobile internet users in China has surpassed 1 billion. It is noteworthy that in the age distribution of Chinese Internet users, the number of netizens aged 6–19 has reached 158 million in China, accounting for 15.7% of the total netizens (China Internet Network Information Center, 2023). That is to say, mobile phones have become the “standard configuration” for many teenagers. Mobile phones have become essential multifaceted tools for teenagers, serving as crucial aids for learning, versatile entertainment platforms, and efficient communication bridges (Wang et al., 2017). However, there is a troubling downside to this widespread adoption. Among adolescents with inadequate self-control, mobile phones can cultivate a growing dependency that may even escalate into mobile phone addiction (MPA) (Lee and Lee, 2017). Mobile phone addiction refers to the behavioral addiction in which an individual’s physical, psychological and social functions are obviously damaged due to excessive use of mobile phones (Liu et al., 2020a,b). This addiction manifests as an obsessive and compulsive need to use mobile phones, often resulting in a variety of debilitating physical and psychological consequences. Studies have shown that excessive and uncontrolled use of mobile phones can significantly impact teenagers’ academic performance and overall quality of life (Samaha and Hawi, 2017). Additionally, it can contribute to a range of mental health issues, such as depression, anxiety, stress, and compromised sleep quality (Stankovi et al., 2021). Given these profound effects, it is crucial to delve deeper into the underlying factors and mechanisms driving MPA among Chinese adolescents. Such exploration holds the key to developing effective prevention and intervention strategies that can mitigate the negative impacts of this growing phenomenon.

The process of gaining knowledge and adapting to society begins within the family, where insufficient parental education, guidance, and supervision can lead to externalized problematic behaviors among adolescents, including MPA (Liu et al., 2020a,b). Within this context, the concept of parental phubbing emerges as a significant factor. This term refers to parents’ preoccupation with their mobile phones while supposedly spending time with their children, often resulting in emotional neglect or overlooking their offspring’s needs (Niu et al., 2020). Prolonged exposure to a detached family environment, over which adolescents have no control, may drive them to seek refuge in their mobile phones, using them as a means of escapism from their surroundings (Hong et al., 2019). Consequently, this increases their vulnerability to developing MPA over time. Drawing from general strain theory, adolescents who experience stressful home environments lacking parental care may resort to unhealthy or maladaptive coping mechanisms to alleviate their distress (Jun and Choi, 2015). Furthermore, studies investigating the intergenerational transmission of behavior emphasize that children within the family unit often imitate the behaviors they observe in their caregivers (Haggerty and Carlini, 2020). In the context of parent–child interactions, excessive mobile phone use by parents emerges as a critical risk factor that can influence teenagers’ mobile phone usage patterns. While previous research has established a positive correlation between parental phubbing and MPA in adolescents, a more comprehensive understanding of the underlying mechanisms remains elusive and warrants further exploration.

### 1.1 The mediating role of deviant peer affiliation

To delve into the factors and internal mechanisms of adolescent MPA more directly entails further investigation into how parental phubbing mediates adolescent MPA. Several empirical investigations have discovered that deviant peer affiliation is a proximal risk factor for problematic behaviors (such as MPA) among adolescents (Yoon et al., 2020). On the one hand, parental phubbing can lead to a decrease in the level of attachment children feel toward their parents. Attachment theory posits that a lack of meaningful bonds between parents and children during crucial stages of development fosters insecure attachments, which in turn, exacerbate deviant behaviors in adolescents. This suggests that when parents are consistently distracted by their phones, it can have a detrimental impact on the emotional security and behavioral outcomes of their children (Bowlby, 1982). As parents fail to give timely attention and feedback to children’s emotional expressions, adolescents would turn to other environments (such as deviant peer affiliation) to meet their psychological needs (Yoon et al., 2021). On the other hand, social learning theory suggests that the behavior of peers acts as an example and reinforces the behavior of adolescents (Grusec, 1992). Besides, deviant peers often have higher social status and are less disciplined by societal rules, so teenagers are more likely to befriend them (Jia et al., 2017). With the increasing popularity of mobile phones, more teenagers, especially delinquent teenagers, use mobile games as the core component of social interaction (Xie et al., 2019a,b). Therefore, under less control of parents on their behaviors, adolescents may easily make friends with deviant peers and tend to overuse mobile phones under their influence, gradually resulting in the development of MPA. As such, this study proposes the hypothesis as follows:

Hypothesis 1: Deviant peer affiliation mediates the relationship between parental phubbing and MPA among adolescents.

### 1.2 The moderating role of sensation seeking

Ecological systems theory highlights that personal development results from the interaction between the environment and the individuals. In a similar vein, the externalizing problematic behavior of adolescents is also affected by the interaction between the external environment and the internal psychological characteristics (Bronfenbrenner, 1997). It implies that adolescents with a personality vulnerability (e.g., high sensation seeking) may exhibit more maladaptive behaviors (e.g., MPA) when exposed to adverse environments (e.g., parental phubbing and deviant peer affiliation) (Wang et al., 2019). Sensation seeking is a personality trait expressed in the generalized tendency to seek varied, novel, complex, and intense sensations and experiences and the willingness to take risks for the sake of such experiences (Zuckerman et al., 1978). This study attempts to examine the integrated effect of the interaction between sensation seeking, parental phubbing, and interaction with deviant peer affiliation on MPA. Previous research has demonstrated that sensation seeking is a crucial predictor of MPA, and individuals with higher sensation seeking have a higher risk of developing MPA (Wang et al., 2018). To our knowledge, no research has specifically investigated the moderating role of sensation seeking in the relationship between environmental risk factors (such as parental phubbing and deviant peer affiliation) and adolescent MPA. However, existing studies have indicated that sensation seeking can exacerbate the negative effects of adverse environments on adolescent behavior. Within the family context, research has demonstrated that sensation seeking intensifies the link between childhood emotional neglect and problematic mobile phone use among Chinese adolescents (Chen et al., 2021). Similarly, in the realm of peer interactions, studies have revealed that adolescents with higher levels of sensation seeking tend to exhibit increased aggression when exposed to deviant peer influences (Wang et al., 2017). To address this research gap, we aimed to explore whether adolescents who score higher on sensation seeking (compared to those with lower scores) are more susceptible to engaging in MPA when confronted with parental phubbing or deviant peer affiliation. As such, this study proposes the hypothesis as follows:

Hypothesis 2: Sensation seeking can moderate the effects of parental phubbing and deviant peer affiliation on MPA. Specifically, individuals with high levels of sensation seeking may be more susceptible to the effect of parental phubbing and deviant peer affiliation, resulting in a stronger impact on MPA. Conversely, for individuals with low levels of sensation seeking, the influence of parental phubbing and deviant peer affiliation on MPA may be less pronounced.

### 1.3 The present study

**Figure 1:**
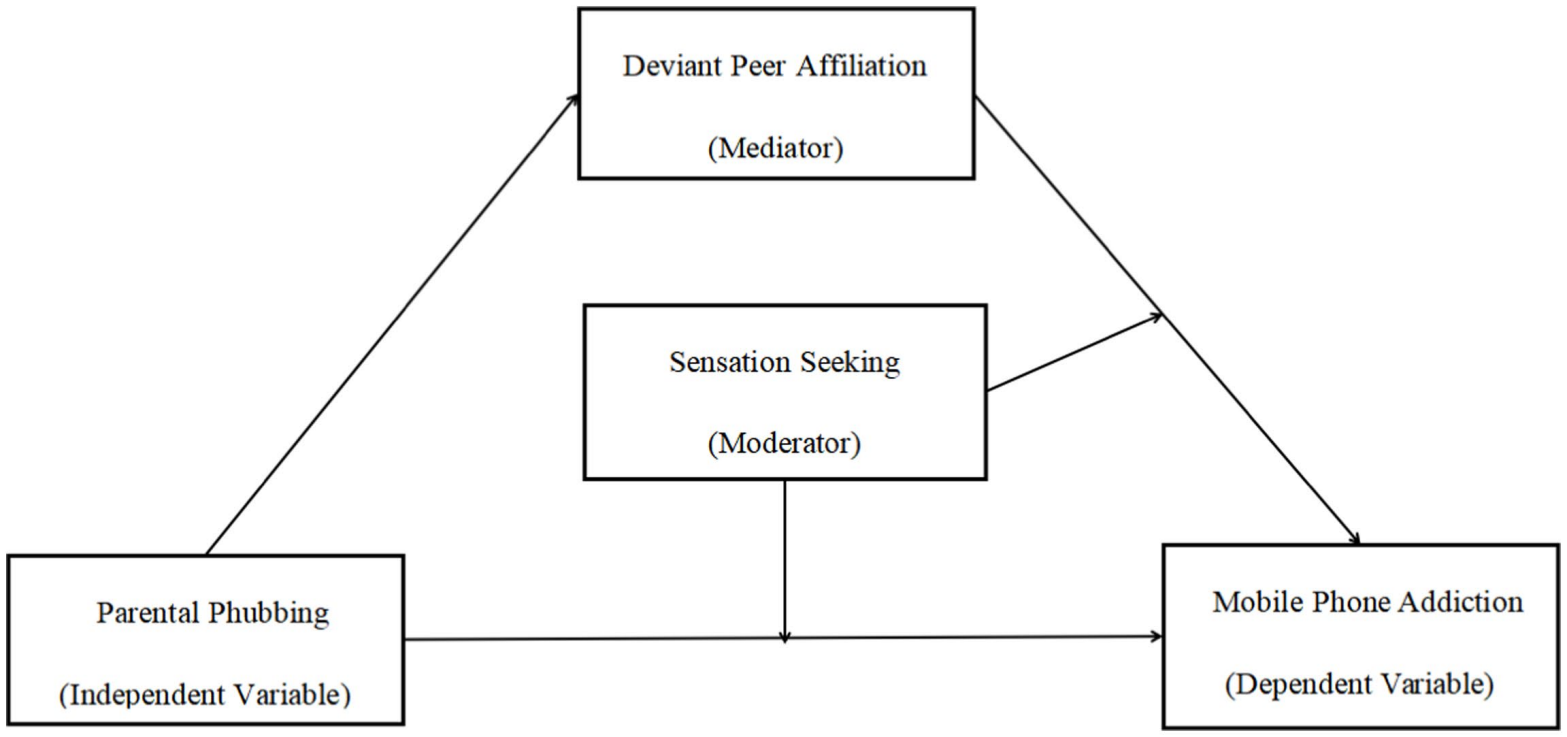
A moderated mediation model of parental phubbing, deviant peer affiliation, sensation seeking and adolescent mobile phone addiction.

As a recently discovered family risk factor, parental phubbing can significantly predict the addictive behaviors of adolescents. Therefore, this research intends to establish a moderated mediation model to clarify the internal mechanism between parental phubbing on adolescent MPA. Based on social learning theories and ecological systems, this paper set out to address the following questions: (1) Whether deviant peer affiliation significantly mediates the effect of parental phubbing on MPA among adolescents; (2) Whether sensation seeking significantly moderates the direct relation and the second path of the indirect relation between parental phubbing and MPA among adolescents. Specifically, the direct and indirect association is stronger in adolescents with high sensation seeking and is weaker in adolescents with low sensation seeking. The proposed model is illustrated in Figure 1.

## 2 Methods

### 2.1 Participants

To enhance the diversity and inclusivity of our sample, we utilized random cluster sampling to enlist participants from three public junior high schools (MPAnning Grades 7 to 9) in Guangdong province, China. Initially, we randomly chose schools to serve as clusters. Subsequently, within each of these schools, we randomly selected classes for survey participation. This approach culminated in a comprehensive questionnaire survey encompassing 820 individuals. Before initiating data collection, we secured informed consent from parents, teachers, and adolescents alike, ensuring that all parties involved were fully aware and agreeable to the nature of the study. Notably, none of the potential subjects declined participation in the survey. Subsequently, 34 entries with missing data were eliminated, with an effective rate of 95.85%. The final sample consisted of 786 adolescents (53.1% males) ranging from 12 to 15 years of age (mean age = 13.71, *SD* = 1.35).

### 2.2 Measures

#### 2.2.1 Parental phubbing

Parental phubbing was assessed using the Parental Phubbing Scale (Xie and Xie, 2020), which was adapted from the Partner Phubbing Scale (Roberts and David, 2016) to assess the students’ perceived parental phubbing. The scale contains 9 items such as “When I have dinner with my parents, he/she will play with the mobile phone.” and “When I go out to play with my parents, he/she will play with the mobile phone.” The participants were requested to answer each item on a 5-point Likert-type scale, from 1 = never to 5 = all the time. All the scores were averaged, and the highest score represented the greatest parental phubbing. It is widely employed among Chinese adolescents, which has good validity and reliability (Liu et al., 2021). A good reliability (Cronbach’s *α* = 0.91) of this scale was found in this study.

#### 2.2.2 Deviant peer affiliation

Deviant peer affiliation was evaluated using the 8-item scale Deviant Peer Affiliation Questionnaire (Li et al., 2016), which has good validity and reliability among Chinese adolescents (Wang et al., 2020a,b; Wang X. et al., 2020). The participants were asked to report how many peers have delinquent behaviors (e.g., smoking, internet addiction, stealing and punishment) on a 5-point scale, from 1 = none to 5 = all the time. The highest scores indicated the highest deviant peer affiliation level. A good reliability (Cronbach’s *α* = 0.92) of this scale was observed in the present study.

#### 2.2.3 MPA

Mobile phone addiction was measured using the Mobile Phone Addiction Index (Leung, 2008). There are four factors of the scale: inability to control craving, feeling anxious and lost, withdrawal or escape, and productivity loss. It contains 17 items, with each item is scored on a 5-point Likert scale. Participants were classified as having had mobile phone addiction if they responded positively to 8 items. Previous research has established that this index is a great measure instrument for Chinese teenagers thanks to its good validity and reliability (Chen et al., 2021). The highest mean represented the highest level of MPA. A good reliability (Cronbach’s *α* = 0.86) of this scale was observed in the present study.

#### 2.2.4 Sensation seeking

Sensation seeking was determined using the Sensation Seeking Questionnaire (Steinberg et al., 2008). This scale contained 6 items. Responses involved a 6-point Likert scale, from 1 = strongly disagree to 6 = strongly agree. The highest score indicated the greatest sensation seeking. This scale has been applied to Chinese adolescents and demonstrated good validity and reliability (Wang et al., 2019). The Cronbach’s α coefficient was 0.89 in the present study.

### 2.3 Covariates

Age, gender, and family socioeconomic status (SES) have been associated with the primary variables used in this study (Peng et al., 2022). Age was calculated based on the participant’s age in years. Gender was represented by a dichotomous variable (female = 0; male = 1). SES was determined based on the mean of 4-item scores (e.g., family per capital monthly income, educational level of each parent, geographical area). Family per capital monthly income was measured by a 10-category variable (1 = ≤¥1,000, 10 = ≥¥9,000).

### 2.4 Procedure

Ethical approval for this study was obtained from the Ethics in Human Research Committee of School of Education, Guangzhou University. After obtaining written informed consent, the questionnaires were distributed, and the information was acquired by well-trained psychology graduates. All participants were allowed to leave a research study at any time. All information were treated in the strictest confidence and used solely for academic research.

### 2.5 Statistical analysis

SPSS 24.0 was employed for descriptive statistics and correlation analysis. Mplus 8.3 was applied to explore the mediating and moderating roles (Muthén and Muthén, 1998). Model fit was evaluated using the multiple fit indices, including comparative fit index (CFI), ratio chi-square over degrees of freedom (*x*^2^/*df*), Tucker–Lewis index (TLI) and root mean square error of approximation (RMSEA). Previous SEM findings suggest that the model fit is considered good when CFI ≥ 0.95, *x*^2^/*df* ≤ 3, RMSEA≤0.05, and TLI ≥ 0.95 (Hayes, 2013). Adolescents’ age, gender, and family SES were statistically controlled as covariates.

## 3 Results

### 3.1 Descriptive statistics

The descriptive statistics and correlation of the study variables are presented in Table 1. Notably, parental phubbing was positively related to MPA, indicating that parental phubbing is a potential risk factor for MPA. Also, parental phubbing was positively correlated with deviant peer affiliation, indicating that adolescents with higher levels of parental phubbing appear to have higher levels of deviant peer affiliation. Moreover, a positive association was observed between deviant peer affiliation and MPA, suggesting that adolescents with higher levels of deviant peer affiliation tend to engage in MPA.

**Table 1:** Means, standard deviations and correlations of the main study variables.

Variables	1	2	3	4	5	6	7
1. Gender	1.00						
2. Age	0.01	1.00					
3. SES	0.03	0.06	1.00				
4. Parental phubbing	0.02	0.06	0.03	1.00			
5. Deviant peer affiliation	−0.02	0.14^**^	0.02	0.53^***^	1.00		
6. Sensation seeking	0.01	0.03	0.03	0.33^**^	0.22^**^	1.00	
7. Mobile phone addiction	0.04	0.05	0.08^*^	0.52^***^	0.59^***^	0.26^**^	1.00
Mean	–	13.17	2.93	3.51	3.61	4.16	3.71
*SD*	–	1.35	0.91	0.87	0.89	0.92	0.86

Gender (1 = male and 0 = female). SES, socioeconomic status. ^*^p < 0.05, ^**^p < 0.01, ^***^p < 0.001.

### 3.2 Testing for mediation effect of deviant peer affiliation

**Figure 2:**
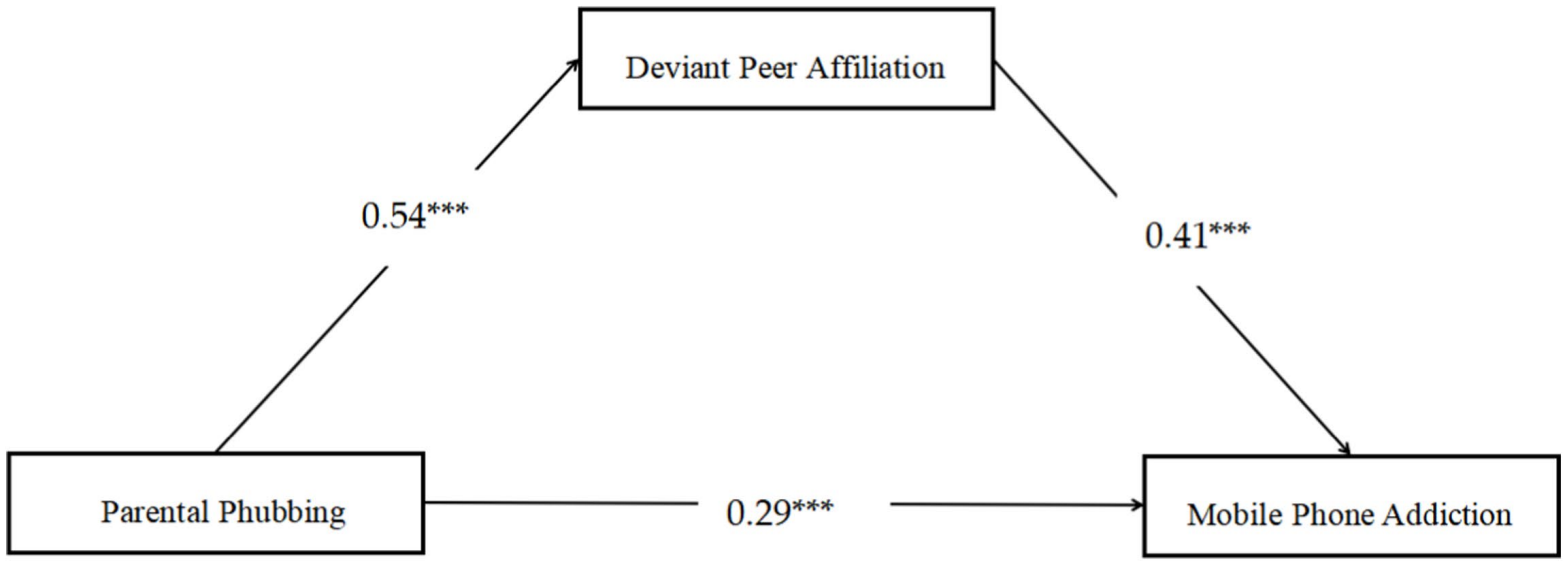
The mediation analysis of deviant peer affiliation in the association between parental phubbing and mobile phone addiction. Standardized regression coefficients are represented by numbers. Paths of gender, age and SES in the model are not displayed. ^***^p < 0.001.

As presented in Figure 2, the mediation model had a good fit for the data: *x*^2^/*df* = 1.75, CFI = 0.99, TLI = 0.99 and RMSEA = 0.02. After adjusting for age, gender and SES, it was observed that parental phubbing could positively predict deviant peer affiliation (*β* = 0.54, SE = 0.03, *p* < 0.001, 95% CI [0.26, 0.50]), and deviant peer affiliation positively predicted MPA (*β* = 0.41, SE = 0.03, *p* < 0.001, 95% CI [0.35, 0.47]). In addition, the residual effect of parental phubbing on MPA was also found (*β* = 0.29, SE = 0.03, *p* < 0.001, 95% CI [0.23, 0.35]). Bootstrapping analysis revealed that deviant peer affiliation remarkably mediated the relationship between parental phubbing on MPA (*β* = 0.22, SE = 0.02, *p* < 0.001, 95% CI [0.18, 0.27]).

### 3.3 Testing for moderated mediation effects

**Figure 3:**
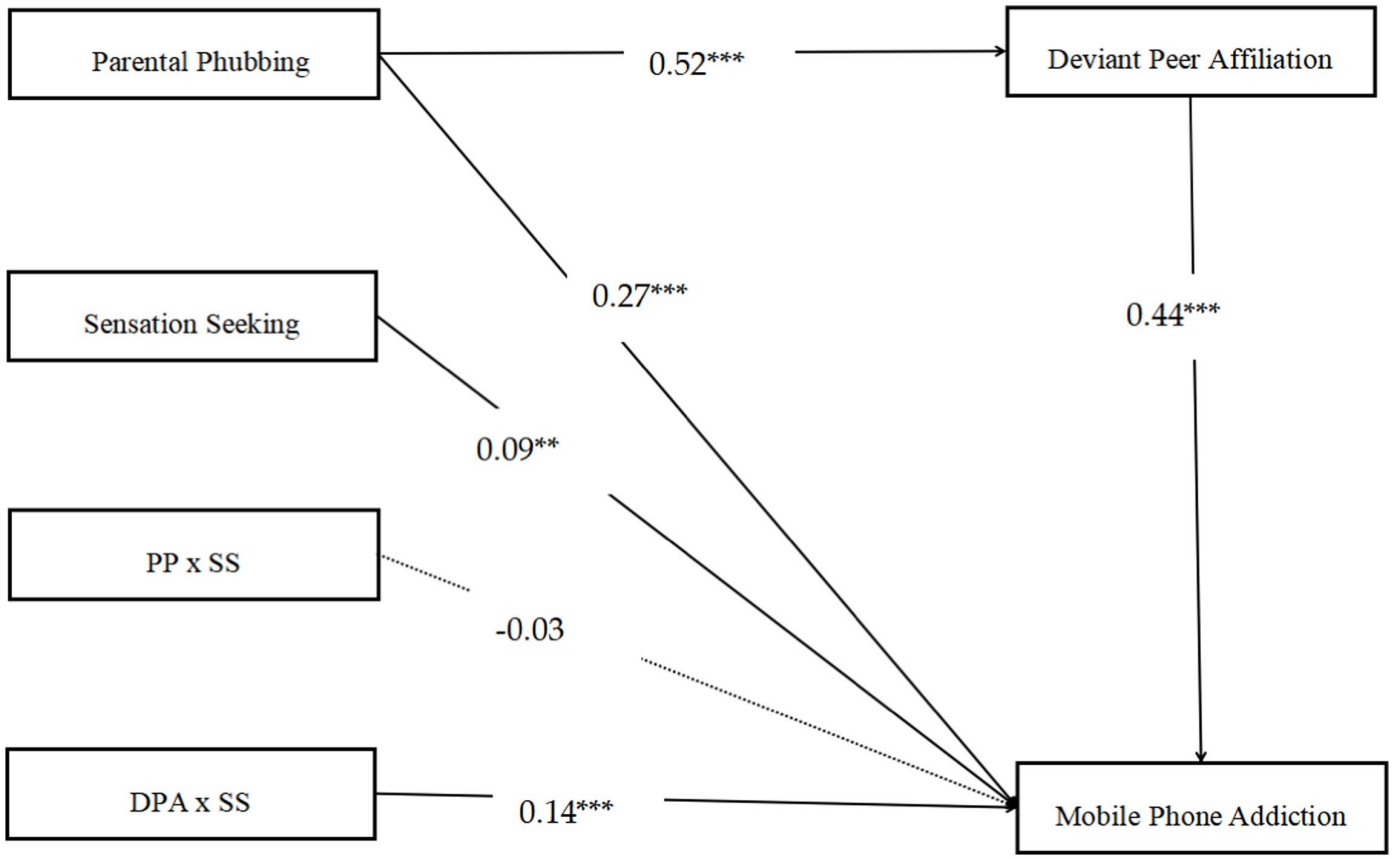
Model of the moderating effects of sensation seeking on the direct/indirect relationships between parental phubbing and mobile phone addiction. Standardized regression coefficients are represented by numbers. Paths of gender, age and SES in the model are not displayed. ^**^p < 0.01, ^***^p < 0.001. PP, parental phubbing; DPA, deviant peer affiliation; SS, sensation seeking.

As displayed in Figure 3, the moderated mediation model had a good fit for the data: *x*^2^/*df* = 2.58, CFI = 0.99, TLI = 0.97 and RMSEA = 0.04. The results demonstrated that sensation seeking did not moderate the direct relationship between parental phubbing and MPA (*β* = −0.03, SE = 0.04, *p* > 0.05, 95% CI [−0.83, 0.41]). Otherwise, it was found that the effect of parental phubbing on MPA through deviant peer affiliation was moderated by sensation seeking. Notably, sensation seeking moderated the correlation between deviant peer affiliation and MPA (*β* = 0.15, SE = 0.04, *p* < 0.001, 95% CI [0.09, 0.21]). Age, gender and SES were included in the regression equation for controlling the covariates.

**Figure 4:**
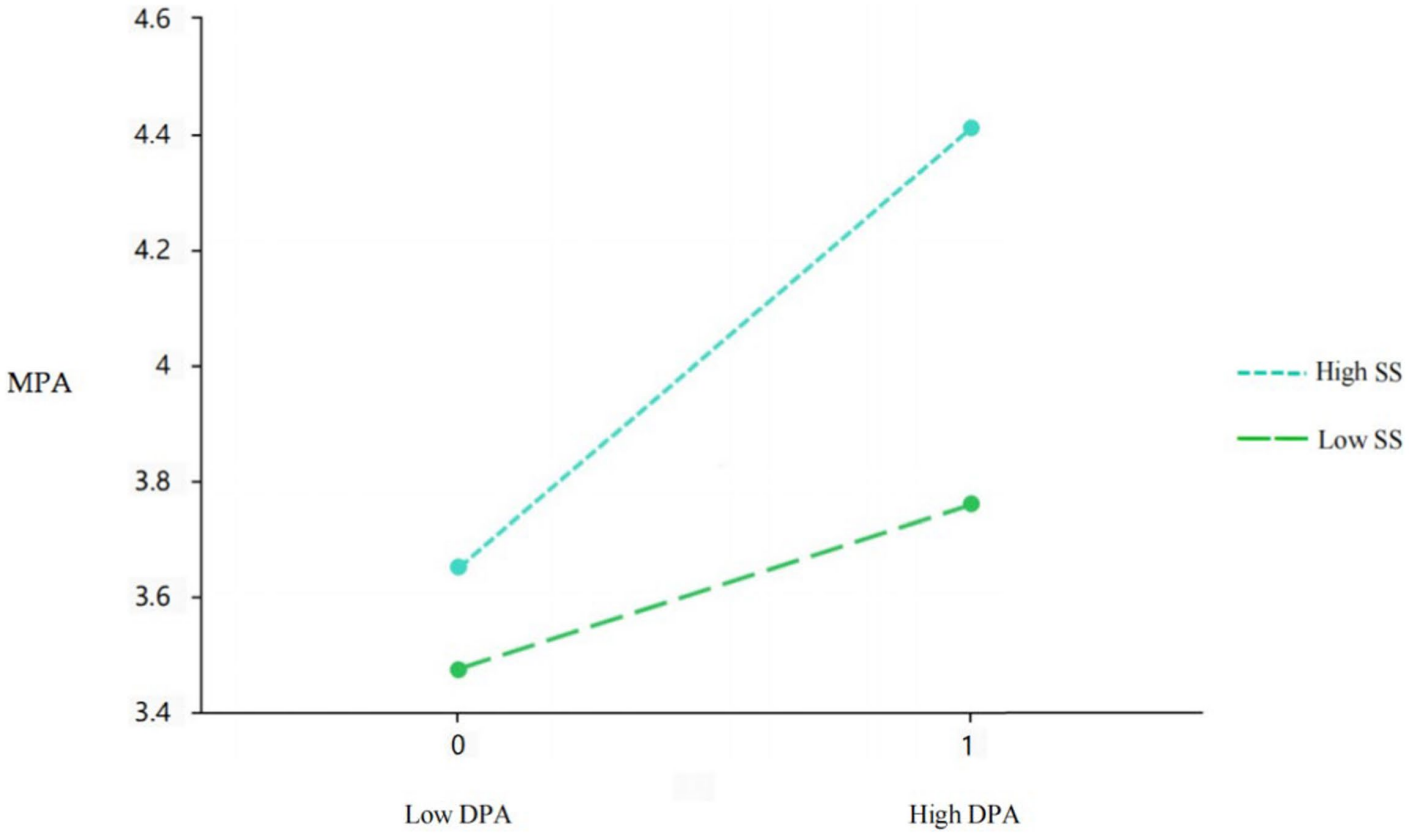
Interaction between deviant peer affiliation and sensation seeking in predicting adolescent MPA. DPA, deviant peer affiliation; SS, sensation seeking.

To further explore the moderating role of sensation seeking, the plots of the relationship between deviant peer affiliation and MPA at 2 levels of sensation seeking (1 SD above and below the mean) are demonstrated in Figure 4. The simple slope test indicated that deviant peer affiliation was positively related to MPA (indirect effect = 0.71, *SE* = 0.04, *p* < 0.001, 95% CI [0.63, 0.78]) in adolescents with high sensation seeking, but the effect was reduced in those with low sensation seeking (indirect effect = 0.42, *SE* = 0.04, *p* < 0.001, 95% CI [0.34, 0.49]).

## 4 Discussion

The current study utilized a moderated mediation model to examine the mediating and moderating mechanisms in the association between parental phubbing and adolescent MPA. The results indicated that the detrimental effect of parental phubbing on adolescent MPA was explained in part by deviant peer affiliation. Moreover, this indirect link was stronger for individuals with higher sensation seeking than for those with lower sensation seeking. These findings provide a more detailed understanding of how and when parental phubbing is related to adolescent MPA.

### 4.1 Parental phubbing and adolescent MPA

This study further confirmed the previous research results (Niu et al., 2020), that is, there is an obvious positive correlation between parental phubbing and MPA among middle school students, meaning that the higher the frequency of parental phubbing, the higher degree of their children’s MPA. According to social learning theory, parents consistently function as role models for their children, communicating specific behavioral patterns, associated attitudes, and societal norms through their actions (Grusec, 1992; Zhang et al., 2021). Adolescents within families are particularly susceptible to observing and emulating their parents’ behaviors. Consequently, when they notice their parents’ attention being drawn to mobile phones, they may follow suit, ultimately culminating in their own struggles with mobile phone addiction (Xie et al., 2019a,b). Moreover, attachment theory posits that a healthy parent–child attachment relationship, founded on mutual trust and communication, can foster a sense of security and parental care among adolescents. This, in turn, can significantly diminish their reliance on mobile phones for emotional support. Conversely, in the absence of such a relationship, adolescents may seek solace in the virtual world of mobile phones to compensate for emotional voids (Zhang et al., 2021). Parental phubbing, unlike traditional forms of parental neglect, exhibits a more specific directionality. That is, parents’ neglect of their children often stems from their engrossment in mobile phones and other digital media (Bai et al., 2020). This behavior can leave children feeling unworthy or less “captivating” compared to the allure of the online world. Over time, this may result in the development of an insecure attachment style between adolescents and their parents, further increasing their vulnerability to mobile phone addiction (Niu et al., 2020).

### 4.2 The mediating role of deviant peer affiliation

The mediation effect analysis revealed that the mediation of deviant peer affiliation on parental phubbing and MPA is significant in adolescents, that is, deviant peer affiliation will catalyze the effect of adverse environmental factors on problematic behaviors in adolescents. This validates the Hypothesis 1 and is consistent with previous finding (Liu et al., 2018). On the one hand, parental phubbing will reduce the energy that parents invest in raising their children, making them unable to provide family warmth and emotional support to adolescents. With insufficient parental emotional warmth and weakened family functions, teenagers would seek social support and emotional belonging from other organizations (including deviant peer affiliation) (Yang et al., 2021). On the other hand, social learning theory suggests that the behavior of peers will act as a role model and reinforcement for individuals (Zhai et al., 2019; Lin et al., 2020), and adolescents who are neglected and ignored by their parents tend to strengthen their identification with the negative environment where they live and even have the idea of “letting things slide” as to learn and imitate the bad behavior of deviant peer groups (Yoon et al., 2020). Moreover, with the increasing popularity of mobile phones, mobile games have become one of the important means of peer communication (especially among deviant peers) (Liu et al., 2020a,b). As adolescents interact more with deviant peers, the risk of developing MPA also increases.

### 4.3 The moderating role of sensation seeking

This study also discusses the individual differences in the mechanism, that is, the moderating effect of sensation seeking. The social-ecological systems theory holds that the mental and physical development of adolescents is affected by the interaction between individuals and the environment (Bronfenbrenner, 1997). Based on the view that individual factors and environmental factors work together, this study examined whether sensation seeking has a moderating effect on the association between adverse environments and adolescent MPA. It is found that sensation seeking is a risk factor for MPA in adolescents, which is in line with what has been found in previous studies (Wang et al., 2019, 2020a,b; Wang X. et al., 2020). However, the present study found that sensation seeking only moderates the mediation effect of deviant peer affiliation on MPA. That is, the negative impact of deviant peer affiliation on adolescent MPA is stronger in adolescents with higher levels of sensation seeking than in those with lower levels of sensation seeking. First, high sensation seekers usually prefer novel stimuli and desire for high arousal experiences and are not mature enough in risk assessment, so in the frequent interaction with deviant peers, they tend to seek thrills and pleasure with their peers while ignoring the risks and harms of the overuse of mobile phones (Wang et al., 2019), consequently leading to MPA. Furthermore, according to the views stated in previous studies, when a risk factor appears at the same time as other risk factors, its cumulative risk effect will produce a more negative impact instead of the simple addition of individual risk effects (Mann et al., 2015). It can be inferred that sensation seeking (risk factor) will “amplify” or “enhance” the negative effect of the interaction with deviant peer affiliation (risk factor) on MPA. If high sensation seekers are meanwhile influenced by deviant peer affiliation, they are more likely to develop MPA.

The present study also found that the direct effect of sensation seeking on the model, that is, the moderating effect of parental phubbing on adolescent MPA, was not significant. Family environment and peer relationship, as two critical micro systems in personal development, can affect the processes of adolescents’ physical and mental growth (Zhai et al., 2019). First, as the micro system that has the greatest impact on individual growth, the family environment and the impact of family function are fundamental to the development of adolescents (Sela et al., 2019). One-year longitudinal studies have shown that the negative parent–child association experienced by individuals in childhood had a long-term profound adverse effect on children’s future behavior. Even if they are no longer traumatized at this stage, their problematic behavior still exists (Taha et al., 2014). Although peer relationships become increasingly important in adolescents’ emotional support as they grow up, the influence of family environment on individuals is still deeply rooted and permanent (Copp et al., 2021). Compared with the stable family environment, deviant peer affiliation, as a varying late-stage environmental variable, is more likely to be affected by more stable individual variables such as sensation seeking in a comprehensive system (Zhai et al., 2019).

### 4.4 Limitations and implications

Several limitations that need to be addressed. Firstly, self-report scales were used to assess all variables, thus, the results might be influenced by some potential bias. Therefore, considering the objective measures of technology use in future research could be more accurate and help avoid interference of potential bias. Second, there might be differences in the influences of the father and the mother on their children’s adaptation (Geng et al., 2021), future studies should focus on these differences. Thirdly, our sample included only adolescents in mainland China, which limited the generalizability of the results. Perceived parental phubbing might be more likely to affect adolescents’ mental health and behaviors in Chinese society (collectivistic society) than in western societies (individualistic societies) (Wang et al., 2020a,b; Wang X. et al., 2020). Thus, further larger population studies are required to improve the generalization of our findings. Fourth, the relationship between parental phubbing and MPA might be influenced by different factors, but only three covariates (age, gender and SES) were included in the moderation-mediated model. Hence, more covariates are needed to improve the applicability of the model.

Despite these limitations, our study demonstrated the moderating and mediating factors of sensation seeking and deviant peer affiliation for the first time, which are responsible for the relationships between parental phubbing and MPA. Moreover, this study is important for the management and prevention of MPA among Chinese adolescents. The results showed that parental phubbing was closely associated with deviant peer affiliation, which exhibited a strong relationship with MPA. Hence, it is recommended that parents can provide more emotional care to their children and improve their parenting ability in order to reduce their children’s deviant peer affiliation, thereby decreasing the risk of MPA in adolescents. Additionally, our findings also verified the moderating effect of sensation seeking on the indirect relationship between deviant peer affiliation and MPA in adolescents. It is worth mentioning that previous studies have shown that sensation seeking can be molded (Martins et al., 2008). Effective intervention on sensation seeking can help reduce individual addictive behavior (Mahu et al., 2015). Therefore, educators should timely identify and screen out individuals with high sensation seeking, and relieve the risk of MPA by intervening and reducing the level of individual sensation seeking.

## 5 Conclusion

In summary, parental phubbing was positively related to adolescent MPA while deviant peer affiliation remarkably mediated the association between parental phubbing and MPA in adolescents. In addition, sensation seeking could obviously moderate the 2nd stage of the indirect path. Notably, adolescents with higher sensation seeking were more engaged in MPA compared to those with lower sensation seeking, under the circumstance of deviant peer affiliation.

## Data availability statement

The original contributions presented in the study are included in the article/supplementary material, further inquiries can be directed to the corresponding author.

## Ethics statement

Ethical approval for this study was obtained from the Ethics in Human Research Committee of School of Education, Guangzhou University. Written informed consent was provided by the participants' legal guardian/next of kin.

## Author contributions

SM: Conceptualization, Data curation, Formal analysis, Funding acquisition, Investigation, Methodology, Project administration, Resources, Software, Supervision, Validation, Visualization, Writing – original draft, Writing – review & editing. XB: Writing – review & editing. HC: Writing – review & editing. YM: Writing – original draft, Writing – review & editing.
